# Supplementary material for: How to improve representativeness and cost-effectiveness in samples recruited through meta: A comparison of advertisement tools
Source: PLoS One. 2023 Feb 8;18(2):e0281243. doi: 10.1371/journal.pone.0281243 (PMC9907806; doi:10.1371/journal.pone.0281243)
Supplement: S1 File — (PDF) [file pone.0281243.s001.pdf]

How to Improve Representativeness and Cost-effectiveness in Samples Recruited Through Meta: A Comparison of Advertisement Tools

Supporting Information

Contents

1 Study **2**

2 Demographic Targeting Across Countries **3**

3 Attention Check **4**

4 Cross-group distribution of participants recruited through conversion **5**

5 Comparison of response quality for targeting strategies **6**

6 Setting-up a conversion campaign on Facebook Ads Manager - A user's guide **6**

6.1 Create and verify a Facebook Pixel . . . 7
6.2 Setting up an event . . . 8
6.3 Verify your domain and register your event on your domain . . . 9
6.4 Creating advertisements . . . 9
6.4.1 Choices at campaign level . . . 10
6.4.2 Choices at ad set level . . . 11
6.4.3 Choices at ad level . . . 12

1 Study

The pre-analysis plan (PAP), the questionnaire, and the revision to the pre-analysis plan submitted after the data collection in the UK can be accessed here https://osf.io/sx7ve. The study received ethical approval by the University of Glasgow's College of Social Sciences Research Ethics Committee on January 24, 2021 (approval number: 400200091).

2 Demographic Targeting Across Countries

Figure S.1: Demographic targeting across countries and recruitment speed

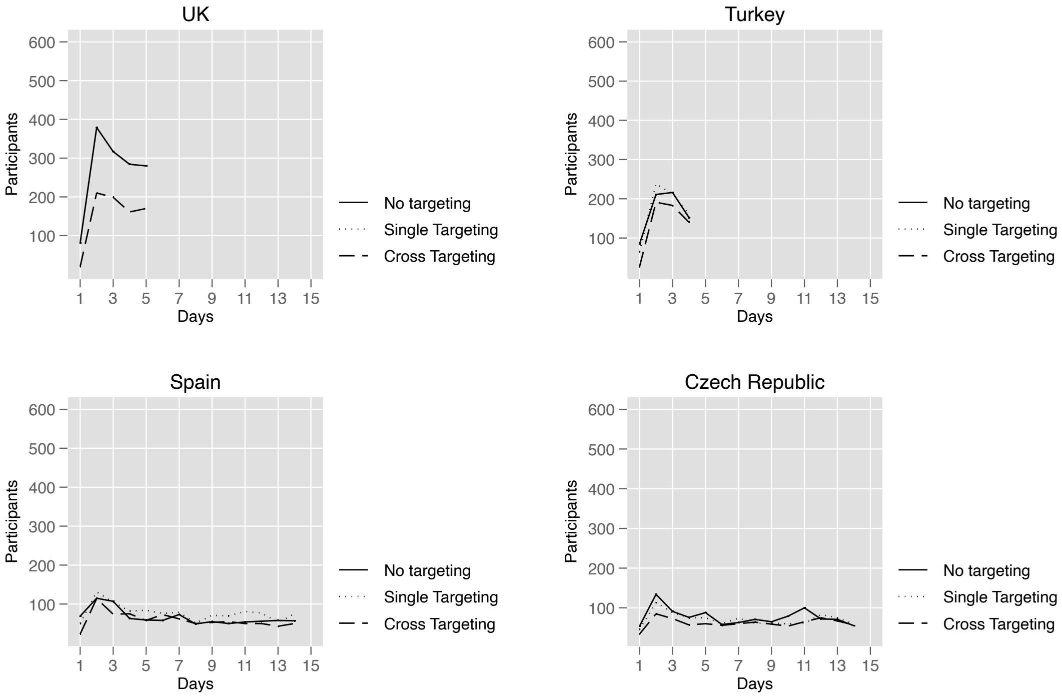

3 Attention Check

Figure S.2: Attention check

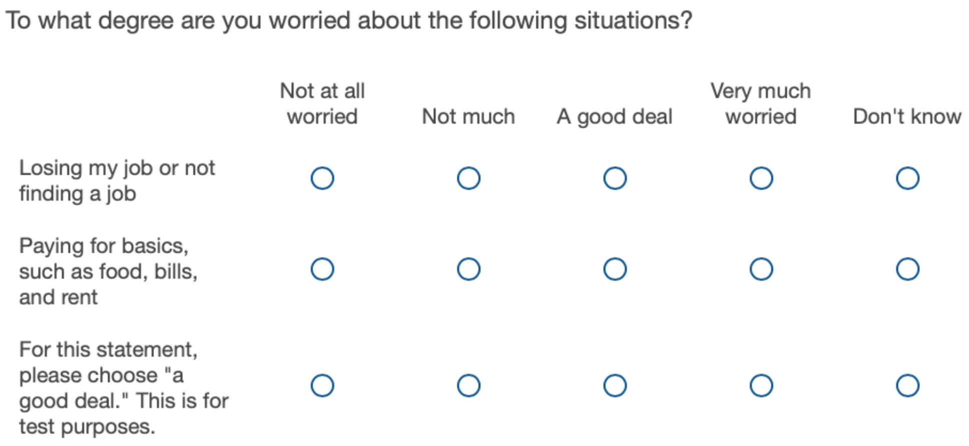

To what degree are you worried about the following situations?

	Not at all worried	Not much	A good deal	Very much worried	Don't know
Losing my job or not finding a job	○	○	○	○	○
Paying for basics, such as food, bills, and rent	○	○	○	○	○
For this statement, please choose "a good deal." This is for test purposes.	○	○	○	○	○

4 Cross-group distribution of participants recruited through conversion

Table S.1: Cross-group distributions of participants recruited through conversion by age, gender and education

Dem.		UK			Turkey		Spain			Czech Republic					
group	None	Cross	None	Single	Cross	None	Single	Cross	None	Single	Cross				
Y, F, NC	**5*** (1%)**	**5*** (1%)**	**8%**	9*** (2%)	11*** (2%)	**18*** (4%)**	**14%**	4*** (1%)	6*** (1%)	**16** (4%)**	**7%**	13 *** (2%)	21*** (4%)	45 (10%)	9%
Y, M, NC	3*** (1%)	**13*** (4%)**	**10%**	6*** (1%)	31*** (5%)	**43** (9%)**	**15%**	5*** (1%)	10*** (2%)	**19** (4%)**	**8%**	84 * (14%)	67 (13%)	26 * (6%)	11%
Y, F, C	**16** (2%)**	5** (1%)	5%	24 (4%)	**34 (6%)**	27 (6%)	5%	4*** (1%)	14** (2%)	**22 (5%)**	**4%**	37 *** (6%)	42*** (8%)	27 *** (6%)	3%
Y, M, C	11*** (2%)	**16 (5%)**	**5%**	38* (7%)	54*** (10%)	**55*** (12%)**	**5%**	6** (1%)	**33** (5%)**	22* (5%)	3%	94 *** (16%)	64*** (12%)	32*** (7%)	2%
M, F, NC	4*** (1%)	**18** (5%)**	**10%**	10*** (2%)	14*** (2%)	**28*** (6%)**	**17%**	25*** (4%)	34*** (5%)	**44 (10%)**	**12%**	17 *** (3%)	17*** (3%)	49 (11%)	14%
M, M, NC	13*** (2%)	**20* (6%)**	**10%**	41*** (7 %)	**52*** (9%)**	50** (11%)	17%	22*** (4%)	37*** (6%)	**53 (12%)**	**13%**	93 (16%)	90 (17%)	42** (10%)	15%
M, F, C	32 (5%)	**35** (10%)**	**6%**	**53*** (9 %)**	**53*** (9%)**	39*** (8%)	3%	48 (9%)	**56 (9%)**	47** (10%)	7%	27 (5%)	27 (5%)	38 *** (9%)	4%
M, M, C	22** (3%)	**25 (7%)**	**6%**	**139*** (24%)**	81*** (14%)	58*** (13%)	4%	39 (7%)	**43 (7%)**	41** (9%)	6%	124 *** (21%)	71*** (14%)	41 *** (10%)	4%
O, F, NC	**62*** (9%)**	53 (15%)	16%	6*** (1 %)	18*** (3%)	**27 (6%)**	**8%**	48*** (9%)	**64*** (10%)**	57** (12%)	18%	11 *** (2%)	27*** (5%)	47 *** (11%)	19%
O, M, NC	**141*** (21%)**	59* (17%)	13%	46 (8 %)	**57 (10%)**	40 (9%)	10%	104** (18%)	**115** (18%)**	47* (10%)	14%	45*** (7%)	37*** (7%)	40** (9%)	14%
O, F, C	**146*** (22%)**	55*** (16%)	4%	51*** (9%)	**55*** (9%)**	35*** (8%)	1%	109*** (19%)	**105*** (16%)**	45*** (10%)	3%	14 (2%)	22*** (4%)	28 *** (6%)	2%
O, M, C	**216*** (32%)**	47*** (13%)	5%	**162*** (28%)**	107*** (19%)	40*** (9%)	2%	**161*** (28%)**	125*** (20%)	46*** (10%)	4%	50 *** (8%)	48*** (9%)	26 *** (6%)	3%
Total (100%)	**671**	351		**585**	567	460		584	**658**	467		629	553	452	

Note: Chi-square goodness of fit comparing Facebook and population samples: * p≤0.05, ** p≤0.01, ***p≤0.001. Age = Young (Y), Middle-aged (M), Old (O). Gender = Female (F), Male (M). Education: Non-college (NC), College (C). Cells highlighted in bold refer to the campaign, which returned most participants in that particular demographic category in comparison to the other campaigns.

5 Comparison of response quality for targeting strategies

Table S.2: Comparison of response quality for targeting strategies

	UK			Turkey			Spain			Czech Rep.		
	NT	ST	CT	NT	ST	CT	NT	ST	CT	NT	ST	CT
Passed attention check	94%	n/a	93%	80%	80%	78%	86%	83%	84%	88%	90%	87%
Median duration (in min.)	6.6	n/a	6.7	8.53	8.58	8.2	7.28	7.65	7.42	6.15	6.5	6.5
Mean duration (in min.)	10.7	n/a	16.2	11.4	10.7	13.3	9.7	11	10	8.63	8.83	23.3
Responded open-ended	96%	n/a	94%	93%	93%	92%	90%	90%	89%	88%	89%	85%
Word count in open-ended	17	n/a	17	13	11	11	14	15	15	13	13	12
Provided contact details	69%	n/a	67%	41%	43%	40%	42%	41%	40%	52%	54%	49%

Note: NT=Non-targeting, ST: Single-targeting; CT: Cross-targeting. Only respondents completing their surveys are included in this table.

6 Setting-up a conversion campaign on Facebook Ads Manager - A user's guide

Conversion campaigns are used to track behavior of Facebook users outside of Facebook environment and optimize Facebook advertisements accordingly. For example, these campaigns allow a business owner to show her Facebook advertisements to Facebook users who have already demonstrated an interest in products sold on her website. Alternatively, conversion campaigns can be used to show advertisements to Facebook users who are similar to people that shop on a business owner's website.

Our research has demonstrated that conversion campaigns can be used to direct Facebook advertisements to Facebook users who are more likely to complete an online survey that is conducted outside of Facebook -we conducted our survey on the Qualtrics survey platform. This became possible as we established a tracking system that allowed us sending data from Qualtrics to Facebook regarding which Facebook users completed our survey. Below we detail steps that we took to establish this tracking system.

Before creating any advertisement campaigns on Facebook, a researcher should first have a Facebook account and a Facebook page affiliated with the account. With permission from the University of Glasgow, we used University of Glasgow's Facebook page to publish our advertisements on Facebook. We would otherwise need to start a new Facebook page. However, creating a new Facebook page can easily be done on the homepage of a Facebook profile.

If a researcher is not planning to use conversion campaigns, she can directly start creating advertisements through Facebook Ads Manager.[1] If she wants to use conversion

[1] https://www.facebook.com/ads/manager

campaigns, on the other hand, she will need to complete several steps to establish the tracking infrastructure that will be used to pass survey participants' information back to Facebook. Some of these steps are necessary: creating a Pixel, installing Facebook Pixel on your website, and creating web events. Other steps aim to increase the performance of advertisements by increasing channels through which visitor data will be transferred to Facebook: verifying domain ownership and using Conversion API. Below, we are detailing how we completed these steps in our own study.

6.1 Create and verify a Facebook Pixel

Facebook Pixel is a code that is unique to an advertisement account and used to transfer visitor data from another website to Facebook through browser cookies. It is necessary to create a Facebook Pixel code and install it to the website, in which actions to be tracked will be taken.

Creating a Facebook pixel code is straightforward. As listed on Facebook's website,[2] you can go to Events Manager, click "Connect Data Sources," select Web, select Facebook Pixel and click Connect, and add your Pixel Name. Facebook also offers an easy setup menu to create a Facebook pixel if you attempt to create a conversion campaign on Facebook Ads Manager without having a Facebook Pixel.

Once Facebook Pixel is created, researcher needs to place this code on the website, in which user behavior will be tracked. Facebook offers three alternatives to add Pixel code to your website, shown in Figure S.3.

Figure S.3: Adding Facebook Pixel to a website

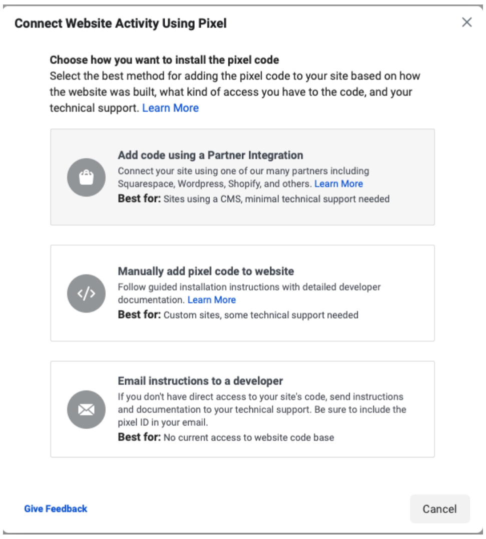

[2] https://www.facebook.com/business/help/952192354843755?id=1205376682832142

For a researcher, the website on which the survey is hosted, i.e. Qualtrics in our case, is the website that needs to be tracked. Yet, we preferred to install Facebook pixel code on a WordPress web page controlled by us rather than on Qualtrics for several reasons. First, our main concern was to distinguish Facebook users that would successfully complete our survey from Facebook users who would visit our Qualtrics page but leave it immediately or after answering a couple questions only. Using a third web page is a secure way of doing this. Second, as detailed below, registering domain ownership on Facebook facilitates more effective uses of Facebook's tracking infrastructure. We are not aware of a way of verifying domain ownership with Qualtrics. Finally, adding code on Qualtrics could only be possible by manually adding code, which would require some technical knowledge,[3] but Facebook's partner integration is available for WordPress, without requiring any coding skills.

Choosing to use a partner integration allows researcher to install Facebook pixel code without any coding. Facebook offers partner integrations for many websites, including WordPress. Once we chose WordPress, Facebook asked us to install a plugin on WordPress, named "Facebook for Wordpress." After installing this plugin, we followed an easy set-up tool, opened as a pop-up window on Wordpress, to link our WordPress page to the specific ad account and Facebook pixel that we used for this advertisement campaign. Completing these steps allowed Facebook to track user behavior taken on our WordPress page.

6.2 Setting up an event

Facebook calls actions taken by website visitors on your website as "events". Facebook can offer optimization towards different events taken on your website, such as purchase, lead, or adding an item to shopping cart. As such, it is necessary to introduce to Facebook different events that your visitors can take on your website. An advertiser can choose one of standard events already defined by Facebook or create a new event. Facebook offers an event setup tool, as shown in Figure S.4.[4]

Of various alternative events defined by Facebook, we picked "view content," and we added the URL address of a web page we had created for this purpose. This was a "Thank You" page, which was shown to survey participants automatically transferred to this web page after successfully completing our survey. This is how we could send data of survey completers from Qualtrics to Facebook.

[3] See instructions here: https://annie-y-chen.com/post/setting-up-facebook-pixel-qualtrics/

[4] Event set up tool automatically follows while connecting a WordPress account to Facebook. Alternatively, researchers can reach event set up tool by choosing Events manager → choose a pixel → settings → event setup

Figure S.4: Setting up an event

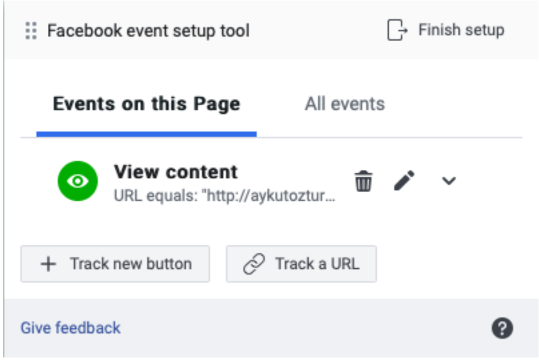

6.3 Verify your domain and register your event on your domain

Verifying Pixel will be enough to start using conversion events, but you might also receive a warning from Facebook when you choose a conversion event that you have just introduced to Facebook: *"This event has not been prioritized for iOS 14.5. Ad sets using this event may not be delivered to people who opt out of tracking on iOS 14.5 or later devices."*

As part of its policy of "protecting customer privacy", Apple has recently started to force apps to receive permission from Apple users to be able to track them. This means that Apple users can now easily opt out of tracking, which makes conversion campaigns less effective[5] To provide tracking data under these circumstances, Facebook offers aggregated event measurement, which requires Facebook users to verify domain ownership of their website and register events on their domain.[6]

Facebook offers three ways for verifying domain ownership, as shown in Figure S.5: adding a meta-tag to your HTML source code, uploading an HTML file to your root directory, and updating the DNS TXT record with your domain registrar. We followed the last way. After visiting to our domain registrar (namecheap.com → domain list → advanced DNS), we added the TXT record provided by Facebook as TXT record.

6.4 Creating advertisements

Once these steps are completed, a researcher will be able to create conversion campaigns, as well as traffic and reach campaigns. To create an advertisement, one goes through three hierarchically organized levels at Facebook advertisement manager: campaign, ad set, and ad. Ad is the lowest level; this is where the content of the advertisement

[5]Early data shows that most of Apple users actually opt out of tracking when this option is offered: https://mashable.com/article/ios-14-5-users-opt-out-of-ad-tracking.

[6]More information is available here: https://www.facebook.com/business/help/721422165168355?id=1877298665783613

Figure S.5: Domain verification in Facebook

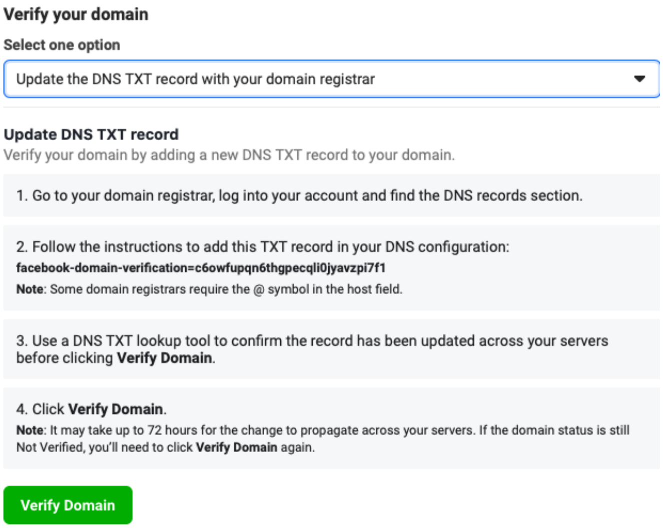

is designed. Ad set is the medium level; it can include a group of advertisements. Demographic targets of advertisements, optimization strategies, and budgets can be determined at the ad set level. By grouping advertisements under ad sets, Facebook facilitates optimization at the advertisement level. In other words, a business can prepare several different advertisements targeting the same audience and then let Facebook's optimization algorithm to determine which of these advertisements works best for that target group and channel resources accordingly. Campaign is the highest level; it can include multiple ad sets, each having different audiences and seperate advertisements. At the campaign level, business owners choose campaign objectives, according to which Facebook offers a menu of optimization and pricing strategies. Below, we detail choices we made in each of these levels, going from campaign to ad set and ad levels.

6.4.1 Choices at campaign level

At the campaign level, one is first required to choose if her ads fall under Special Ad Categories, which include social issues, elections, or politics. This is required by Facebook "to enable healthy discourse and increase transparency and accountability." Advertisements under this category are subject to more detailed checks and procedures. Although our survey covered these issues, our Facebook advertisements did not. Most of our advertisements did not mention about politics at all. For these reasons, we did not list our advertisements under the Special Ad Categories.

Facebook offers a series of campaign objectives to choose one from. Below, in Figure S.6 eleven campaign objectives that were offered by Facebook in November 2020, when our study was designed, can be seen:[7]

[7] As we were preparing this document, Facebook announced some changes in campaign objectives.

Figure S.6: Campaign objectives offered by Facebook

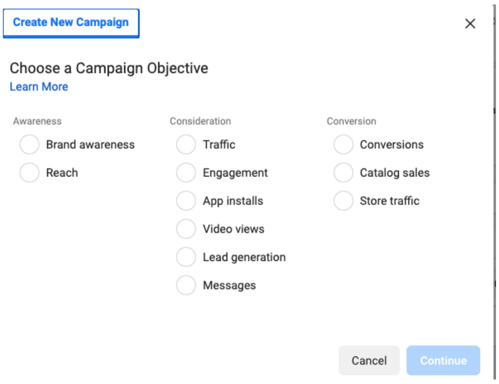

There are several tools that we did not use at the campaign level. We did not use A/B Test offered by the Facebook Ads Manager (FAM). A/B tests can be used to test how variations of campaigns, ad sets, or ads work in comparison to each other. We also did not set a spending limit at the campaign level; we set a spending limit at the ad set level.

6.4.2 Choices at ad set level

Options offered at the ad set level might differ based on the campaign objective. Conversion objective offers "conversion" as an optimization strategy, in addition to optimization strategies offered at the traffic category. We chose "conversion" as our optimization strategy for this objective. We chose a one day click conversion window, instead of seven days window. This is because we expected users who saw our ad to complete the survey on the same day. As a result, Facebook optimized advertisement delivery by using data from conversions that happened within one day of interactions with our ad.

Conversion objective also offers setting cost caps and bid caps. Thus, one can prevent Facebook algorithm from spending more money for a conversion than the value of that conversion for the advertiser. We did not use cost and bid caps.

For ad sets, one can either choose a daily budget or a lifetime budget. A daily budget sets a daily spending limit for Facebook; while lifetime budget lets Facebook to spend all the budget in the optimum way. We opted for a daily budget for each ad set so that we could closely follow the performance of advertisements each day and stop the delivery of advertisements once we we reached enough number of participants. In our study, each ad set included only one advertisement so these spending limits were also spending limits at the ads level.

Although there will be changes in objective names, they will still have the same functions. For more information, see https://www.facebook.com/business/help/325793898950394

Figure S.7: Building a URL parameter

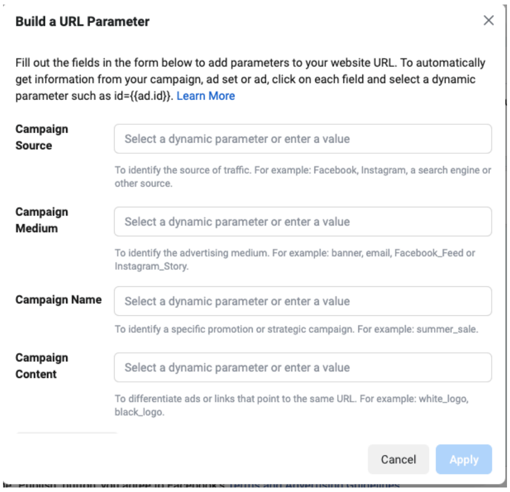

We used a variety of demographic targeting in our study. FAM is usually successful at targeting for age and gender categories, while education targets are less accurate. Facebook allows including or excluding participants based on their education level and offers a long list of education levels, based on how people indicate their highest education levels.

FAM offers a wide range of placement alternatives. One can determine whether the advertisements are shown at mobile or desktop devices. One can choose on which platform advertisements will be shown: Facebook, Instagram, Audience Network, and Messenger. Finally, FAM asks the location of advertisements in each social media page: feeds, stories, in-stream, search, messages, in-article, or apps and sites. For all of these options, one can simply choose “automatic placement” so that FAM allocates the ad set’s budget based on where they are more likely to perform better. We chose “automatic placement” and let Facebook decide the placements of our advertisements.

6.4.3 Choices at ad level

Choices at the ad level are the same for the three campaign objectives, i.e. traffic, reach, and conversion. We created advertisements using an image, primary text, headline, description, website URL, and display link, as shown in Figure **??**. We added a “call to action” button with words “learn more”.

In terms of format, Facebook offers single image or video, carousel (2 or more scrollable images), and collection (group of items that open into a fullscreen mobile experience). All of our advertisements fell under the first category, i.e. single image or video. Finally, to track which advertisements people were coming from, we added parameters to our survey page’s URL link, using the form shown in Figure S.7.

After completing all of these steps, clicking on “Publish” button submit the advertisement campaign to Facebook’s review, which takes around several hours. Once the advertisement is online, it is advised to follow its performance through Facebook Ads Manager.
