# Supplementary material for: How to improve representativeness and cost-effectiveness in samples recruited through meta: A comparison of advertisement tools
Source: PLoS One. 2023 Feb 8;18(2):e0281243. doi: 10.1371/journal.pone.0281243 (PMC9907806; doi:10.1371/journal.pone.0281243)
Supplement: Attachment — Submitted filename: Response Letter.pdf [file pone.0281243.s002.pdf]

Dear editor and reviewers,

Thank you very much for the feedback you provided for our work. All of the suggestions we received were useful, and we did our best to implement them. We believe that these changes have significantly improved our manuscript. We have also made some other changes to follow Plos One's stylistic requirements. Below we list these changes.

Response to the First Reviewer

Reviewer 1 is right that starting conversion campaigns is more complicated than starting other advertisement campaigns. We tried to be clearer about this point in the revised manuscript. In the conclusion part, between lines 461 and 464, this point is emphasized one more time. It is important to add that there is no reason for an advertiser to be unable to start a conversion campaign as long as she is well-informed about the required steps. In addition to the Online Appendix, we have also published an online handbook to help researchers with these steps.

Reviewer 1 suggested us to revise the structure of our paper. We made significant changes in the manuscript following this suggestion. First, we restructured our manuscript following Plos One guidelines. We separated "Meta Advertisement Tools" and "Materials and Methods" sections more clearly, removing all references to our study in the former section. Finally, we simplified the entire text and removed all parts that were repetitive or secondary. These helped us to shorten the paper.

We added the time period of data collection to Methods section; you can see these between lines 126 and 134. Exact dates of all studies are also available in Table 2 in page 8.

Our reference to poststratification weights in the original submission might have been confusing. We did not generate any poststratification weights in this study; we only used weights provided by ESS and CSES to weigh nationally representative surveys. Based on survey codebooks, we know that both of these weights are constructed using demographic information. We removed references to poststratification weights from the main text to be less confusing. Our explanation on this issue, along with the name of the variables used, can be found on page 11, in the footnote of Table 3.

Following Reviewer 1's advice, we spent more time discussing the limitations of our study in the conclusion section. We believe that the biggest limitation of our study is "temporal validity." Given the rapid change in online advertisement tools, it is hard to say how long our findings will keep their relevance. We had already mentioned "temporal validity" in our original submission. We now moved this part to the Conclusion to emphasize it, and we further developed our discussion. Please see lines 471 to 492 for these discussions.

We have added the proportion of Facebook users in our cases to the main text. You can find these numbers at lines 141 to 146.

Following Reviewer 1's suggestion, we removed Figure 2 from the main text. Unfortunately, editorial assistants at Plos One informed us that we were not allowed to include the image of an

advertisement due to copyright issues. For this reason, we had to drop Figure A.1. from the paper. We are now directing our readers to a handbook, in which this image is shown.

We have updated Table 1 to be more informative and less confusing. We believe that the table is helpful for readers to visually see combinations of campaign objectives and targeting tools; we have added information about which combinations are used in each of the countries.

Finally, we have also checked the entire text again for typos and other grammatical mistakes.

Response to the Second Reviewer

Reviewer 2 encourages us to better emphasize some of the most important findings from our research. Following his/her suggestions, we have enriched our summary in the last paragraphs of the Introduction part. Please see lines 44 to 63 in the final manuscript to see this revised part. Reviewer 1 also asks very good questions about whether we can develop any cost-effective strategies based on these findings. Unfortunately, our data does not allow us to answer these questions.

Reviewer 2 asks us to be more explicit about the fact that the Facebook platform is (1) non-transparent and (2) constantly changing. This is an extremely important issue as Facebook is about to introduce new changes to campaign objectives. We have revised our conclusion part taking this comment into account. In two separate paragraphs we are now discussing these limitations.

Reviewer 2 was critical about the way our paper ended. Following with this criticism, we reorganized our conclusion part. We are now starting with the discussion of findings and limitations and end with a broader picture on the promises of online tools and the necessity to produce more work exploring these tools.

Following Reviewer 2's advice, we added the number of respondents to the final row in Table 4.

Following Reviewer 2's advice, we aligned all cells in Table 3 and 6. We believe this made these tables more readable.

We have also corrected all other grammatical errors pointed out by Reviewer 2.

Response to Journal Requirements

We have made some changes in the manuscript to fit with Plos One's style guidelines. We carried the entire text to a Plos One template hosted on Overleaf. We removed all footnotes in the main text. Some of the footnotes were moved to the main body of the article. Other footnotes were transferred to the Online Appendix. We deleted the footnotes that did not have direct contributions to the paper.

We added more details to the main text about the participant consent. These details can be seen at line 194-201 at the final version of our manuscript.

We want to clarify that this paper was published only as a pre-print before; no parts of it has been published as a peer-reviewed article.

Figure 1 does not include copyrighted material. We have created this map in Stata, using publicly available information.

We have removed Figure A.1. from our submission due to copyright issues.

We reviewed the reference list one more time. We do not have any retracted study in our reference list. However, we made some changes to the list. We have removed works that are no more cited in the final manuscript, and we added some new studies to the reference list that are now cited in the final manuscript.

Apart from these changes, we have replaced most of the references to “Facebook” with “Meta,” as a result of the company’s recent brand change.
