# Supplementary material for: How to improve representativeness and cost-effectiveness in samples recruited through meta: A comparison of advertisement tools
Source: PLoS One. 2023 Feb 8;18(2):e0281243. doi: 10.1371/journal.pone.0281243 (PMC9907806; doi:10.1371/journal.pone.0281243)
Supplement: Attachment — Submitted filename: Response to reviewers.docx [file pone.0281243.s003.docx]

**RESPONSE TO REVIEWERS**

We are grateful that both the editor and the Reviewer 2 went through our manuscript carefully and pointed out some sufficient language in our manuscript. We corrected these mistakes, and the manuscript is in a better form now. Furthermore, we have also had the entire manuscript proofread by a professional proofreader. Below we list all the changes we made during the second revision process.

**Editor comments:**

*1: Editor: "Please report the languages used in the survey experiments and how this may have affected survey responses."*

We have conducted all of the surveys in official and the most spoken languages in those countries. This has increased the size of our audience in each country. This practice is also the norm in the political science discipline.

In response to this comment, we have added a new sentence on page 4: “All of the surveys were conducted in the official and the most spoken language of the respective country, i.e., English, Turkish, Spanish, and Czech, allowing us to maximize the size of our audience.”

**Reviewer 2’s comments:**

1: First, Reviewer 2 points out a series of language mistakes, which have now all been corrected.

2: *“On p3.66, the authors state that "the former is usually not even more expensive than the latter" - I would specify "in our experience", because it is hard to conclude this more generally.”*

We have added “in our experience” to the referred sentence.

3: “*Meta is misquoted on p3.83; this does not match the text in the source.”*

Thank you for noting this problem. We have realized that Meta had made a change to the URL link. We have updated the URL link.

4: *“On p4.110, "you can" sounds a bit too casual to me.”*

Instead of “you can see,” we have inserted “please see.”

5: *“The text refers to Turkey as a "non-democratic country"; while it is not an open society, I believe that factually (technically) it is considered a democracy. Similarly, the paper implies that Spain is between the UK and Turkey in terms of political openness, but is this true? In my understanding it would be as open/democratic as the UK.”*

According to V-Dem and Freedom House classifications, which are used by most of the political scientists, Turkey is currently ruled by an electoral authoritarian regime. On the other hand, the reviewer is right that today’s Spain is as democratic as the UK, although democratic institutions are younger in this country. We have revised the relevant paragraph to focus on the level of economic development in Spain and the Czech Republic and add a reference to Varieties of Democracy dataset:

“The UK constitutes a typical case of an advanced democracy, and it is the second most-studied country in political science according to Wilson (2020). However, many researchers are increasingly using Meta advertisements to study less developed, non-democratic countries, where data collection is more restricted. Turkey represents such a context. It is a middle-income country that was, according to the influential Varieties of Democracy dataset, an electoral authoritarian regime by the time our study was conducted. Spain and the Czech Republic are younger democracies that are located between the UK and Turkey in respect of their levels of gross domestic product (GDP) per capita. The Czech Republic is also distinguished by its relatively small population, which amounts to only 10 million people.”

6: *“On p5.162 you say that "campaigns from the same country stayed open for the same amount of time", but Figure 2 suggests otherwise.”*

We believe that we were not clear enough in our sentence. What we meant is that each of the campaigns run in a country stayed open for the same amount of time. Thus, for example, all campaigns in the UK stayed open for 5 days and all campaigns in Turkey stayed open for 4 days. This is what Figure 2 shows as well.

To make this clearer, we revised the sentence. It now reads: “each of the campaigns running in a country stayed open for the same amount of time.”

7: *“I think Table 2 would be clearer if the left column (A/B/C) were dropped and the italicized entries were called "click-through rate" and "completion rate". Then, the note at the bottom of the table could explain how these were calculated.”*

We made the changes in Table 2 as suggested by Reviewer 2.

8: *“On page 18, the description of the weights is missing some punctuation/explanatory words.”*

Page 18 included the bibliography in the previous manuscript. Yet, we used to refer to weights in page 11, saying: “We weighted these benchmarks with weights included in these surveys. We used pspweight for ESS E1010 2 for CSES. Both of these weights are constructed based on demographic information.” We believe this is what the reviewer means.

We have updated this sentence to make it clearer. The new version reads: “We weighted population estimates from these surveys with weight variables provided in the original survey. We used *pspweight* variable for ESS survey and *E1010*_2 variable for CSES survey. According to codebooks of these surveys, both of these weight variables were constructed based on demographic information.”

9: *“On p9.378, the table says that participants recruited through conversion spent more time on average, but this is only true in 2/4 contexts.”*

Reviewer 2 is right in pointing out that in half of the countries participants recruited through traffic spent more time on average. We revised the sentence accordingly. The new sentence reads: “As Table 4 shows, however, there is no clear winner in this regard.”
